# Editorial: Systems biology and single-cell analysis of cancer metabolism and its role in cancer emergent properties

**DOI:** 10.3389/fonc.2023.1217212

**Published:** 2023-05-31

**Authors:** Dongya Jia, Xuefei Li, Yapeng Su

**Affiliations:** ^1^ Center for Theoretical Biological Physics, Rice University, Houston, TX, United States; ^2^ CAS Key Laboratory for Quantitative Engineering Biology, Shenzhen Institute of Synthetic Biology, Shenzhen Institutes of Advanced Technology, Chinese Academy of Sciences, Shenzhen, China; ^3^ Program in Immunology & Herbold Computational Biology Program, Fred Hutch Cancer Center, Seattle, WA, United States

**Keywords:** systems biology, single cell analyses, cancer metabolism, metabolic plasticity, mathematical modeling

Cancer cells can reprogram their metabolic activities to adapt to heterogeneous tumor microenvironments and to survive various treatments, referred to as metabolic plasticity (1, 2). The past two decades have witnessed our advanced understanding in how cancer cells can acquire multiple metabolic phenotypes, such as glycolysis, oxidative phosphorylation (OXPHOS), the hybrid metabolic phenotype and the metabolically “low-low” phenotype (2, 3). The different cancer metabolic phenotypes are associated with varying cancer metastatic (2) and drug-tolerant potentials (4–6). To decipher the sheer complexity and the multi-faceted nature of cancer metabolism, systems biology approaches that emphasize the interactions between genes, proteins, and metabolites have been developed to create a synergy between theoretical/computational and experimental biology and have led to discoveries at a rapid pace.

Recent advancement in technologies have provided researchers with tools to rigorously quantify cancer metabolism, particularly at the single-cell and the subcellular level. In this Research Topic, Wei et al. reviewed the recently developed technologies for single-cell metabolomics measurement and the integration of multi-omic measurements. To analyze the rapidly increasing cellular metabolism data, Ng et al. provided a summary of the open-source python-based computational toolboxes. Through integrating newly developed technologies with computational toolboxes, we acquired a better understanding of individual metabolic genes and metabolites. Fung et al. used non-invasive Raman-based optical imaging techniques to conduct the 3D spatial and chemometric analyses of triple negative breast cancer (TNBC) cells under the tandem modulation of two key metabolites – insulin and methionine. The authors observed altered *de novo* lipogenesis, 3D lipid droplet morphology, and lipid peroxidation under various methionine and insulin concentrations and verified significant interaction of insulin and methionine metabolism. Altered fatty acid metabolism is often associated with cancer development (7). Wang et al. provided a review of Acetyl-CoA Carboxylases (ACC), the first rate-limiting enzyme in fatty acid synthesis, covering its structural feature, regulatory mechanism, and roles in cancer development and other diseases. The authors highlighted the regulation of ACC by AMPK and PI3K/Akt/mTOR pathways and its role in post-translational modifications (e.g., acetylation). In addition to ACC, other metabolic genes have been identified for prognosis. Liu et al. showed that the PLA2G2D expression exhibits a positive correlation with immune cell infiltration and favorable immune checkpoint blockade therapy. Chedere et al. showed that nicotinamide adenine dinucleotide (NAD) can classify hepatocellular carcinoma (HCC) patients into three categories depending on their nicotinate phosphoribosyltransferase status, providing a better resolution in understanding the heterogeneity of HCC patients. As we acquire a better understanding of metabolites and metabolic pathways, we may intervene and suppress cancer metabolism. Hou et al. reviewed the current state of arginine deprivation and replenishment therapies for glioma, and the authors emphasized the importance of assessing cancer metabolic state to ensure the effectiveness of deprivation therapy.

In addition to the advancement of experimental technologies and data analysis methods, mathematical modeling approach has been widely used to elucidate the mechanisms underlying cell-fate decision-making in metabolism. Yu and Wang applied the landscape and flux theory to identify the normal and disease states emerging from a core gene regulatory network, during the development of intestinal-gastric cancer. Yu and Wang elucidated the key regulations that are essential for the transition between normal and disease states. To evaluate therapeutic strategies for melanoma treatment, Hodgkinson et al. applied a data-driven multi-dimensional mathematical modeling approach to simulate melanoma response to different therapeutic strategies – combination therapy, continuous therapy, adaptive therapy. Hodgkinson et al. showed that the order in combination therapy matters and that different therapeutic strategies can lead to different tumor heterogeneity. Furthermore, in the computational analysis conducted by Pillai et al., the authors showed that the dedifferentiation of melanoma cells is accompanied by upregulation of mesenchymal genes, but not a concomitant loss of an epithelial program. Interestingly, progression along the mesenchymal axis correlates with the downregulation of OXPHOS, while glycolytic capacity is largely maintained. The dedifferentiation of melanoma cells is closely-linked with its resistance to BRAF inhibitors (8).

Single-cell analysis of cancer metabolism has been a fast-evolving field. With the increasing capacity of acquiring richer single-cell multi-omics data at a better resolution, it is important to leverage the systems biology approaches to make sense of the data and to decode the mechanisms underlying cancer metabolism and its coupling with other cancer hallmarks. With a better understanding of cancer metabolic plasticity, therapies targeting cancer metabolic dependency in principle can be made more effective.

## Author contributions

All authors listed have made a substantial, direct, and intellectual contribution to the work and approved it for publication.
